# Degradation Investigation of Electrocatalyst in Proton Exchange Membrane Fuel Cell at a High Energy Efficiency

**DOI:** 10.3390/molecules26133932

**Published:** 2021-06-28

**Authors:** Jie Song, Qing Ye, Kun Wang, Zhiyuan Guo, Meiling Dou

**Affiliations:** 1 State Key Laboratory of Advanced Transmission Technology, Global Energy Interconnection Research Institute Limited Company, Beijing 102209, China; yeqing@geiri.sgcc.com.cn (Q.Y.); guozhiyuan@geiri.sgcc.com.cn (Z.G.); 2 Beijing Key Laboratory of Electrochemical Process and Technology for Materials, Beijing University of Chemical Technology, Beijing 100029, China; 2018200475@mail.buct.edu.cn

**Keywords:** proton exchange membrane fuel cell, high energy efficiency, durability, degradation, Pt/C catalyst

## Abstract

The development of high efficient stacks is critical for the wide spread application of proton exchange membrane fuel cells (PEMFCs) in transportation and stationary power plant. Currently, the favorable operation conditions of PEMFCs are with single cell voltage between 0.65 and 0.7 V, corresponding to energy efficiency lower than 57%. For the long term, PEMFCs need to be operated at higher voltage to increase the energy efficiency and thus promote the fuel economy for transportation and stationary applications. Herein, PEMFC single cell was investigated to demonstrate its capability to working with voltage and energy efficiency higher than 0.8 V and 65%, respectively. It was demonstrated that the PEMFC encountered a significant performance degradation after the 64 h operation. The cell voltage declined by more than 13% at the current density of 1000 mA cm^−2^, due to the electrode de-activation. The high operation potential of the cathode leads to the corrosion of carbon support and then causes the detachment of Pt nanoparticles, resulting in significant Pt agglomeration. The catalytic surface area of cathode Pt is thus reduced for oxygen reduction and the cell performance decreased. Therefore, electrochemically stable Pt catalyst is highly desirable for efficient PEMFCs operated under cell voltage higher than 0.8 V.

## 1. Introduction

Proton exchange membrane fuel cells (PEMFCs), as clean and efficient energy conversion devices, have attracted much attention for their promising application in transportation and stationary power plants [1,2]. However, the large-scale commercialization of PEMFCs is still hindered by the high operating costs, mainly due to the low fuel economy and unsatisfied durability [3,4,5,6]. Fuel cell energy efficiency is defined as the ratio of output power to the consumed hydrogen enthalpy. For low temperature PEMFCs, the theoretical energy efficiency is about 83%, calculated by dividing the high heating value of hydrogen (286 kJ mol^−1^) by the Gibbs free energy of fuel cell reaction (237 kJ mol^−1^). Currently, the favorable operation conditions of PEMFCs are with single cell voltage between 0.65 and 0.7 V, corresponding to energy efficiency lower than 57% [7]. For the long term, PEMFCs need to be operated at higher voltage to increase the energy efficiency and thus promote the fuel economy for transportation and stationary applications. To this end, the U.S. Department of Energy (DOE) has set the technical target of fuel cell systems and stacks for transportation application, with peak energy efficiency increased to 65% in 2020 and 70% in the future [8], respectively. The Japanese government also released the strategic road map for fuel cells by reaching energy efficiency of over 55% in about 2025 and over 65% in the future for stationary applications. Accordingly, PEMFCs should demonstrate the capability of working at a single cell voltage higher than 0.8 V.

The lifetime of PEMFCs is also critical for commercialized applications. For transportation, the lifetime needs to meet the 5000 h target and for stationary power, 40,000 h lifetime is required [9]. In recent years, much efforts have been devoted to the durability investigation of PEMFCs under dynamic operations to mimic the transportation drive cycle [10,11,12,13,14,15], including idling [16,17], load-cycling [18,19,20], and start-up/shut-down operation [21,22,23]. Wang et al. [19] conducted a 900 h duration of accelerated stress test on PEMFC using the drive cycle test protocol developed by Chinese NERC Fuel Cell & Hydrogen Technology, and results showed that the cell performance declined by more than 10% with an average voltage decay rate of 70 μV h^−1^. It was proposed that the degradation of key materials in PEMFCs is generally caused by the presence of elevated or fluctuating potential at electrodes under dynamic operations [24,25,26,27,28]. Lin et al. [23] adopted an in-situ segmented cell testing technology to analyze the degradation mechanism of the membrane electrode assembly during start-up and shut-down cycles, and results showed that the performance of fuel cells significantly decreased after 1800 cycles.

With the energy efficiency increased, PEMFCs work at higher voltage, and this brings the challenge for their durability. Although there are many investigations on PEMFC durability, the electrochemical stability of the state-of-the-art materials is still unclear when operated with cell voltage higher than 0.8 V. In this work, PEMFC single cell durability was investigated by operating it at an energy efficiency of over 65%. The performance degradation and the material deterioration were investigated. It was shown that the electrode catalyst was corroded electrochemically and the reaction activity was decreased by over 13% after the 64 h operation. Results indicate that the current catalyst support is not durable for highly efficient PEMFCs and more robust Pt support is needed.

## 2. Results and Discussion

### 2.1. Durability Test of PEMFC

**Figure 1:**
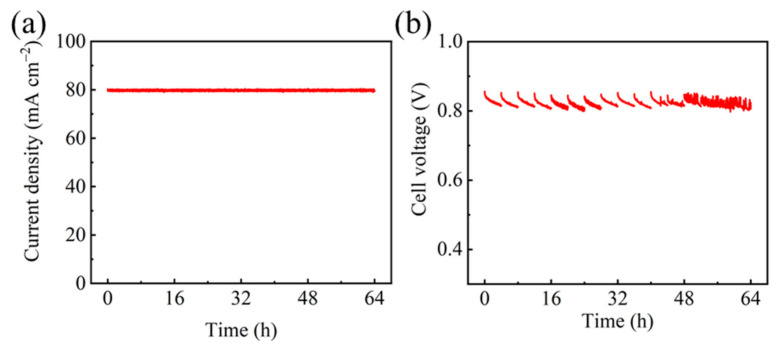
(**a**) Current density versus time and (**b**) cell voltage variation versus time under the current density of 80 mA cm^−2^.

To mimic the operation of PEMFC at a cell voltage higher than 0.8 V, so that the efficiency exceeds 65%, the operating current density was set at 80 mA cm^−2^. The durability of PEMFC was then evaluated by constant-current operation for 64 h (Figure 1a). The performance of PEMFC was monitored by measuring the polarization curves every 4 h. In Figure 1b, the cell voltage showed slight fluctuation due to the disturbance caused by the polarization measurement. After each measurement, the voltage decayed from about 0.85 V to 0.81 V, probably due to the re-balance of water content in PEMFC.

**Figure 2:**
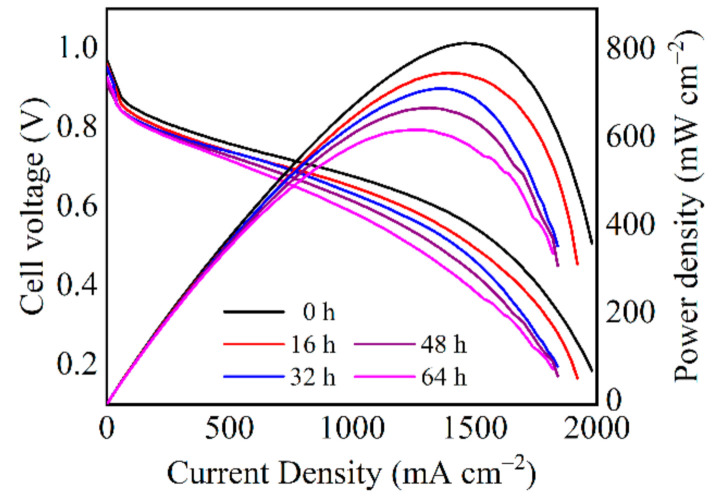
Polarization curves during the durability test.

**Figure 3:**
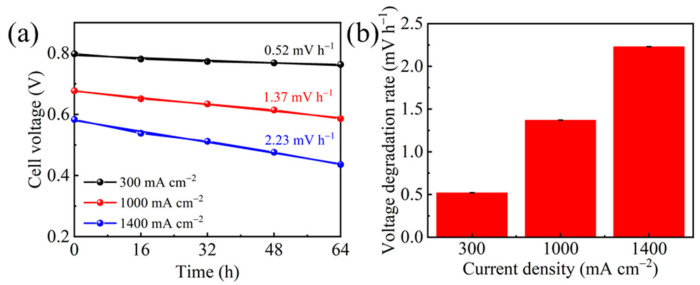
(**a**) Cell voltage versus operation duration and (**b**) the voltage degradation rate at different current densities.

The polarization curves profiled during the operation were shown in Figure 2 to analyze the whole-range performance. Although the cell voltage at 80 mA cm^−2^ displayed little change after the 64 h operation, PEMFC performance decreased significantly at the current density above 1000 mA cm^−2^. The cell voltage dropped by 91 mV at 1000 mA cm^−2^, corresponding to 13.4% performance loss. Considering the short operation duration of only 64 h, the degradation is remarkable, indicating the material deterioration within the cell. Further investigation of the polarization curve indicates that there are increased kinetic, ohmic, and mass transfer loss. The voltage degradation rate was calculated at 300, 1000, and 1400 mA cm^−2^ to demonstrate the performance loss severity at various region. As shown in Figure 3, more significant voltage loss and sharper decay rate were found at higher current density.

**Figure 4:**
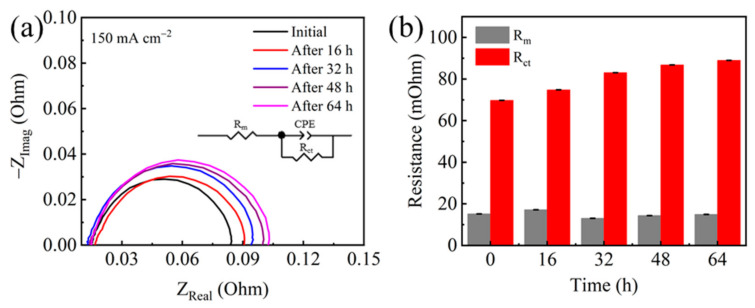
(**a**) Nyquist plots and (**b**) equivalent circuit fitting results at 150 mA cm^−2^ before and after durability test.

To determine the type of polarization loss, electrochemical impedance spectroscopy (EIS) measurement was carried out at the current density of 150 mA cm^−2^ (Figure 4). By fitting the Nyquist plots, the membrane resistance and charge transfer resistance were obtained. Results show that a slight change was observed for the membrane resistance (Table 1), indicating that the change of proton exchange membrane is negligible during the 64 h test. The charge transfer resistance was found to be increased with the elapse of testing time. After the 64 h test, the charge transfer resistance increased from the initial value of 69.76 mOhm to finally 88.97 mOhm (Table 1), suggesting that the apparent catalytic activity was reduced during the durability test probably due to the loss of Pt active sites.

**Figure 5:**
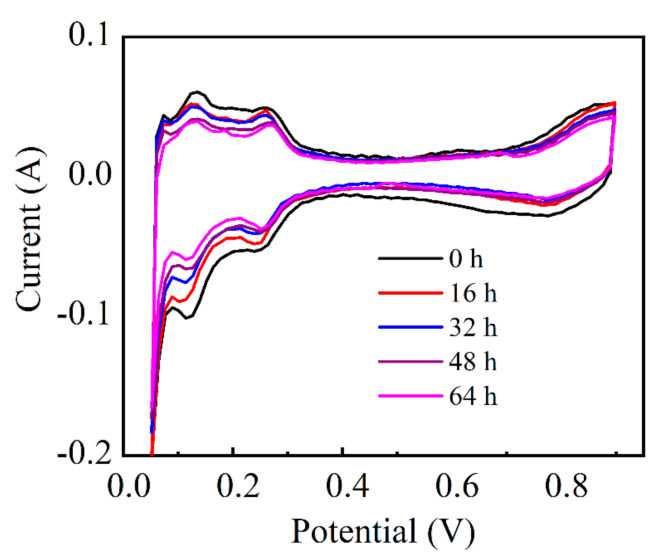
CV curves profiled during the 64 h durability testing.

**Figure 6:**
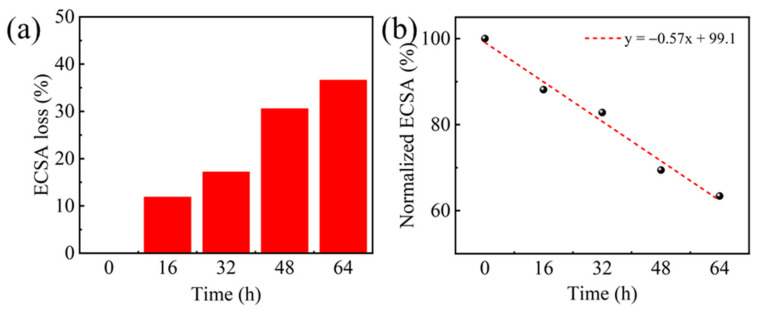
(**a**) Normalized ECSA loss measured during the 64 h test; (**b**) Linear fitting of normalized ECSA loss with operation duration.

To investigate the degradation of Pt/C electrocatalyst, we performed the CV measurement with the scan rate of 50 mV s^−1^ for PEMFC cathode to estimate the cathode electrochemical surface areas (ECSAs, unit: m^2^ g^−1^_Pt_). The value of ECSA indicates the amount of available active sites in the catalyst layer for the oxygen reduction reaction in the cathode. Large ECSA is favorable for a high performance cathode. The ECSAs were calculated by measuring the hydrogen desorption charge integrated from the corresponding potential region in CV curves assuming 0.21 mC cm^−2^ as the charge that is required for oxidizing the hydrogen monolayer on Pt. For comparison, all the calculated ECSAs at different test times are normalized by the initial value. As shown in Figure 5, the corresponding current densities for both the desorption of adsorbed hydrogen and the reduction of Pt-Ox both gradually decrease with the elapse of the testing time, indicating the reduction of ECSAs during the durability test. Therefore, the effective Pt active sites in the cathode were decreased under the high-voltage operation condition. A further investigation indicates that the normalized ECSAs can be linearly fitted with the operation duration. As shown in Figure 6, an average degradation rate of 0.57% h^−1^ was deduced for cathode ECSA.

### 2.2. Physicochemical Characterization of the Cathodei Catalyst Layer

**Figure 7:**
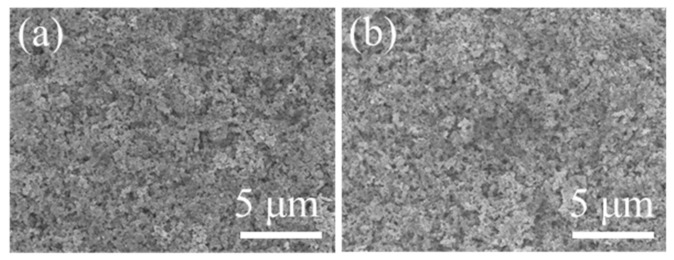
SEM images of the cathodic catalyst layer surface (**a**) before and (**b**) after 64 h durability test.

The durability of the catalyst layer is critical for the lifetime of the fuel cell because the catalyst layer is heart component in membrane electrode assembly for the fuel cell stack. Significant degradation of the catalyst layer leads to the increase in the kinetic polarization loss, irreversibly affecting the durability of fuel cells [29]. Therefore, physicochemical characterizations were carried out to investigate the degradation mechanism of the catalyst layer under the durability test. SEM images show that the cathodic catalyst layer displays a relatively smooth surface distributed with the Pt/C catalyst before the test (Figure 7). After 64 h operation, the surface of the cathodic catalyst layer becomes rougher than the initial catalyst layer, with some collapsed pores in some regions. It implies that the operation at a high voltage has a negative effect on the microstructure of cathodic catalyst layer due to the presence of high potential, and also indicates that the current key materials in fuel cells are not durable.

**Figure 8:**
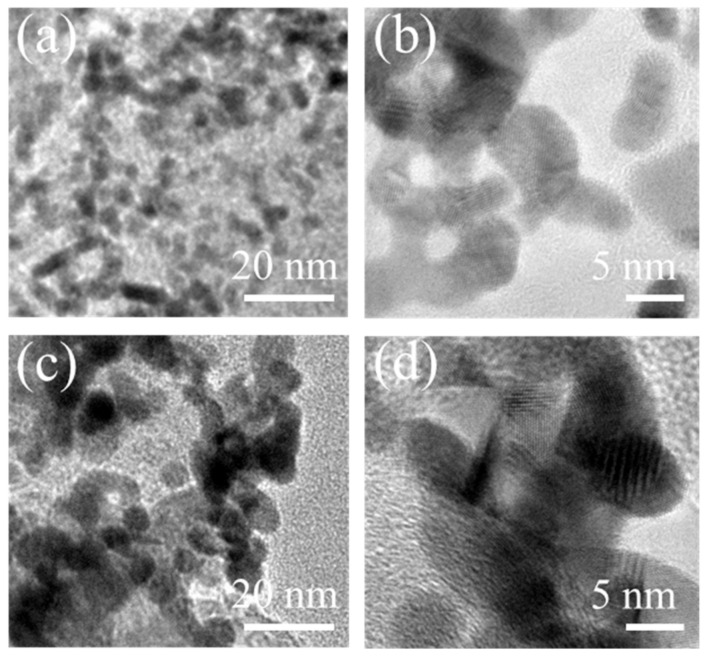
TEM images of cathode Pt/C catalyst before (**a**,**b**) and after (**c**,**d**) the 64 h test at a current density of 80 mA cm^−2^.

TEM characterization was performed to observe the microstructure change of the cathodic catalyst layer before and after the test (Figure 8). TEM images show that the Pt nanoparticles in the Pt/C electrocatalyst mostly exhibit a spherical morphology with an average particle size of approximately 4.0 nm before the test. After the 64 h test, Pt nanoparticles aggregate seriously with an enlarged particle size of approximately 5.4 nm. The obvious growth of Pt nanoparticles is consistent with the change of ECSAs and form CV curves, further confirming that the Pt utilization decreases under such high working cell voltage. Furthermore, the morphology of these Pt nanoparticles changes to be irregular and complex in comparison to their initial morphology, showing significant agglomeration of Pt nanoparticles after the 64 h test. This results reveals that a serious degradation of the Pt/C catalyst in the cathode occurs, which is due to the lasting presence of high potential above 0.8 V. It probably causes corrosion of the carbon support in the Pt/C catalyst, and then leads to the loss of Pt reactive sites, that is, Pt nanoparticles fall off from the carbon surface and agglomerate with an enlarged particle size. Therefore, the effective Ptreactive sites are reduced and ultimately result in a decline in the electrocatalytic activity and thus a decrease in the fuel cell performance.

**Figure 9:**
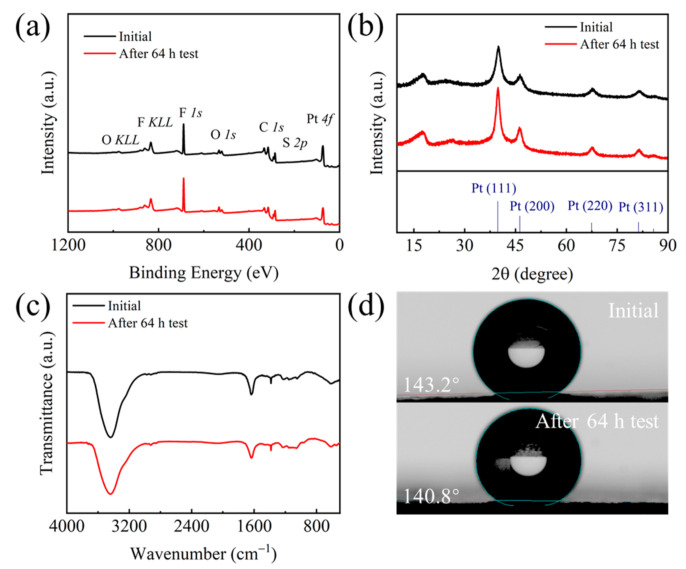
(**a**) XPS survey spectra, (**b**) XRD patterns, and (**c**) FTIR spectra of the cathodic catalyst layer before and after the 64 h test. (**d**) Contact angle test of the cathodic gas diffusion layer before and after the 64 h test.

To investigate the change of surface element composition and content on the cathodic catalyst layer, XPS measurement was conducted for the sample before and after the durability test (Figure 9a). XPS spectra show the presence of C, Pt, F, S, and O on the surface of the cathodic catalyst layer for both before and after the 64 h test, indicating that the main component for the cathodic catalyst layer is the Pt/C catalyst and Nafion ionomer. Further investigations showed that the Pt content decreases significantly after the 64 h test, from 4.75 at% to 3.72 at% (Table 2). The decrease of Pt content after the durability test further indicates that Pt nanoparticles might detach from the carbon support due to the corrosion of carbon under high potential, which is consistent with the TEM results.

XRD patterns were conducted for the membrane electrode assembly before and after the 64 h test (Figure 8b). Results show the diffraction peaks at 39.76°, 46.24°, 67.45°, and 81.29° are assigned to the (111), (200), (220) and (311) planes, respectively, of face-centered cubic (fcc) structured Pt (JCPDS No. 04-0802). The peaks at 18.10° and 26.60° are corresponding to the polytetrafluoroethylene (PTFE) (100) and C (002) plane, respectively, which also confirms the main component of Nafion ionomer and Pt/C catalyst in the catalyst layer. After the 64 h test, no other crystal structure was observed in the membrane electrode assembly. However, the Pt (111) peak becomes sharper than the initial sample, indicating the larger crystalline size of Pt after the durability test, which is in agreement with the TEM results. FTIR test shows that the peaks at 1383, 1231, and 1158 are assigned to the stretching vibrations of S=O bond, asymmetric C-F bond, and symmetric C-F bond (Figure 8c), respectively, confirming the presence of Nafion ionomer. After the 64 h test, no obvious change was observed for the infrared characteristic peaks, indicating that the Nafion ionomer in the catalytic layer did not change obviously. The contact angle test results indicated that the contact angle of the cathodic gas diffusion layer changes negligibly after the 64 h test (from 143.2° to 140.8°) (Figure 8d), suggesting that the durability test did not affect the hydrophobicity of the gas diffusion layer.

## 3. Materials and Methods

### 3.1. Fuel Cell Test

A PEMFC with an active area of 5 cm^2^ was tested by a Scribner 850e fuel cell test station from Hephas Energy Co., Ltd. The catalyst-coated membrane (CCM) sample purchased from the Shaoxing Junji Energy Technology Co., Ltd., Shaoxing, China, in 2019 was fabricated by coating the commercial Pt/C catalyst on Nafion 212 membrane and then combined with two gas diffusion layers to form the membrane electrode assembly. The Pt loading in the anode and cathode catalyst layer is 0.2 and 0.6 mg cm^−2^, respectively, and the fuel cell temperature was set at 80 °C. The flow of hydrogen (H_2_) and the air was controlled at 200 and 800 mL min^−1^, respectively, with the back pressure of 100 kPa and gas relative humidity (RH) of 100% for both anode and cathode. Electrochemical impedance spectroscopy (EIS) test was performed by applying a sine wave distortion (AC perturbation) of 10% DC current amplitude under the galvanostatic mode (frequency range: 10 kHz-100 mHz) to record the impedance spectra with air in cathode and H_2_ in the anode. Cyclic voltammetry (CV) test was conducted to characterize the change in the loss of Pt sites with N_2_ in cathode and H_2_ in anode at the scan rate of 50 mV s^−1^.

### 3.2. Material Characterization

The morphology of the sample was characterized by the scanning electron microscope (SEM, JEOL FE-JSM-6701F) and the transmission electron microscopy (TEM, JEM-2100, JEM-2010F, JEOL, Tokyo, Japan). The surface element composition and content was characterized by X-ray photoelectron spectroscopy (XPS) (Thermo Fisher Scientific ESCALAB 250) using an Al K*α* source. The crystal structure of the sample was characterized using a D/max-2500 X-ray diffraction diffractometer (XRD) equipped with Cu Kα radiation. Fourier transform infrared (FTIR) spectra were recorded on a Nicolet 6700 FTIR spectrophotometer in the range 400–4000 cm^−1^.

## 4. Conclusions

In summary, we have evaluated the durability behavior of a single PEMFC at a high energy efficiency (over 65%) that operates at a voltage higher than 0.8 V and elucidated the material degradation as characteristic. The cell performance decays with a rate of ~1.37 mV h^−1^ at a current density of 1000 mA cm^−2^ during the 64 h test. The decisive factor for the durability deterioration of PEMFC is the degradation of Pt/C catalyst in cathode, showing an obvious growth of Pt nanoparticles with significant Pt aggregation under this operation due to the lasting presence of high potential. This work indicates that the current catalyst is not sufficiently durable for highly efficient PEMFCs, and thus, the exploration of a robust catalyst that is more resistant to a high potential is significantly urgent.

## Figures and Tables

**Table 1:** Fitting results of EIS using equivalent circuit and the ECSA results.

Test Time (h)	Rm (mOhm)	Standard Error	Rct (mOhm)	Standard Error	ECSA (m^2^ g^−1^)	ECSA Loss (%)
0	15.19	0.000102	69.76	0.00046	28.6	0
16	17.18	0.00014	74.80	0.00064	25.2	11.9
32	13.06	0.00011	83.07	0.00067	23.7	17.2
48	14.36	0.00012	86.80	0.00073	19.9	30.6
64	14.93	0.000096	88.97	0.00056	18.2	36.6

R_m_: membrane resistance; R_ct_: charge transfer resistance; CPE: constant phase angle element.

**Table 2:** Elemental composition and content before and after the 64 h test.

Element	C at%	O at%	F at%	Pt at%	S at%
Initial	49.05	8.24	36.21	4.75	1.75
After 64 h test	49.58	9.48	35.37	3.72	1.84
